# Transformation of Human Mesenchymal Cells and Skin Fibroblasts into Hematopoietic Cells

**DOI:** 10.1371/journal.pone.0021250

**Published:** 2011-06-22

**Authors:** David M. Harris, Inbal Hazan-Haley, Kevin Coombes, Carlos Bueso-Ramos, Jie Liu, Zhiming Liu, Ping Li, Murali Ravoori, Lynne Abruzzo, Lin Han, Sheela Singh, Michael Sun, Vikas Kundra, Razelle Kurzrock, Zeev Estrov

**Affiliations:** 1 Department of Leukemia, University of Texas MD Anderson Cancer Center, Houston, Texas, United States of America; 2 Department of Biostatistics, University of Texas MD Anderson Cancer Center, Houston, Texas, United States of America; 3 Department of Hematopathology, University of Texas MD Anderson Cancer Center, Houston, Texas, United States of America; 4 Department of Diagnostic Radiology, University of Texas MD Anderson Cancer Center, Houston, Texas, United States of America; 5 Department of Investigational Cancer Therapeutics, University of Texas MD Anderson Cancer Center, Houston, Texas, United States of America; University of Pittsburgh, United States of America

## Abstract

Patients with prolonged myelosuppression require frequent platelet and occasional granulocyte transfusions. Multi-donor transfusions induce alloimmunization, thereby increasing morbidity and mortality. Therefore, an autologous or HLA-matched allogeneic source of platelets and granulocytes is needed. To determine whether nonhematopoietic cells can be reprogrammed into hematopoietic cells, human mesenchymal stromal cells (MSCs) and skin fibroblasts were incubated with the demethylating agent 5-azacytidine (Aza) and the growth factors (GF) granulocyte-macrophage colony-stimulating factor and stem cell factor. This treatment transformed MSCs to round, non-adherent cells expressing T-, B-, myeloid-, or stem/progenitor-cell markers. The transformed cells engrafted as hematopoietic cells in bone marrow of immunodeficient mice. DNA methylation and mRNA array analysis suggested that Aza and GF treatment demethylated and activated *HOXB* genes. Indeed, transfection of MSCs or skin fibroblasts with *HOXB4, HOXB5,* and *HOXB2* genes transformed them into hematopoietic cells. Further studies are needed to determine whether transformed MSCs or skin fibroblasts are suitable for therapy.

## Introduction

Prolonged thrombocytopenia and delayed immune reconstitution are major causes of morbidity and mortality in patients with hematologic malignancies and life-threatening non-malignant hematologic disorders. Platelet transfusion has reduced hemorrhagic death rate, and white blood cell transfusion has improved the survival of patients with neutropenia-related opportunistic infections [1], [2]. However, multi-donor platelet transfusions occasionally induce platelet refractoriness caused by anti-human leukocyte antigen (HLA) alloimmunization [3] and multi-donor white blood cell transfusions often cause life-threatening acute lung injury [4] and infections transmitted by leukocytes carrying pathogens such as cytomegalovirus [1]. Severe complications limit the HLA-matched donor pool. Repeated apheresis platelet donations adversely affect thrombopoiesis and bone mineralization, and apheresis granulocyte donations occasionally induce inflammatory reactions, thrombocytopenia, bleeding, splenic rupture, capillary leak syndrome, and hepatocellular injury [5]. Thus, an alternative, preferentially autologous, source of hematopoietic cells is needed.

Somatic cells of an adult organism are thought to arise from an irreversible sequential differentiation process in which undifferentiated cells gradually transform into terminally differentiated tissue-specific cells [6]. However, several studies have demonstrated that cells of one type are capable of transforming into cells of another type [7], [8], [9], [10]. For example, hematopoietic cells have been shown to give rise to multiple types of non-hematopoietic cells [11], [12], [13], [14], [15], neuronal cells to hematopoietic cells [16], and dermal cells to neuronal cells, musculoskeletal cells, and adipocytes [17]. Studies in human hematopoietic stem cell transplantation (HSCT) patients support these observations [9], [18]. Donor-derived skin, liver- and gastrointestinal tract tissue-specific cells were detected in biopsy tissues from blood or bone marrow HSCT recipients months after transplantation [19]. However, contradictive data suggested that plasticity in adult stem cells does not occur at an appreciable rate and, thereby, lacks any in vivo developmental or physiological significance [20].

Several cellular reprogramming experiments have been conducted during the past four decades. Somatic cell nuclear transfer (SCNT) was performed in the 1960s [8], [21] and the generation of induced pluripotent stem (iPS) cells capable of forming cells of different tissues has been reported in recent years [22], [23], [24], [25], [26], [27], [28], [29]. Alternative strategies to convert one cell type into another directly, without the need to first revert to an undifferentiated state, such as conversion of dermal fibroblasts and retinal epithelial cells into muscle-like cells [30], [31], [32] or pro-B cells [33] or of inner ear support cells into auditory hair cells [34] have been reported during the past two decades. Recently, pancreatic mature exocrine cells were reprogrammed into functional insulin-producing beta cells by inserting three transcription factors [35].

In addition to direct gene manipulation, tissue reprogramming has also been achieved by pharmacological means. For example, the demethylating agents 5-azacytidine (Aza) and 5-aza-deoxycytidine, now known to inhibit DNA methyltransferases [36], [37], [38], [39], were used to induce differentiation of embryonic cells into muscle cells and adipocytes [40], [41] and of pre-B lymphoma ABLS 8.1 cells into macrophages [42]. DNA methylation is a biochemical modification that, in human cells, primarily affects cytosines when they are part of the symmetrical dinucleotide CpG. Methylation of promoter-associated CpG islands is essential for maintaining the genes' silenced state. DNA methyltransferases induce CpG island methylation. Aza inhibits DNA methyltransferases, and as a result, activates silenced (methylated) genes [36], [37], [38], [39]. Because Aza is routinely used in clinical practice [39], [43] and was successfully used to transform cells of one lineage into cells of another lineage [40], [41], [42], we sought to determine whether Aza could transform easily accessible cells such as human mesenchymal stromal cells (MSCs) into hematopoietic cells.

Mesenchymal cells are easily accessible, and a small number of bone marrow or blood MSCs can be easily expanded [44]. Furthermore, allogeneic mesenchymal cells do not appear immunogenic in non-human primate [45] and human sibling-donor recipients [46], [47], [48]. Indeed, clinical trials with allogeneic mesenchymal cells have been successfully conducted in recent years [47], [49], [50], [51], [52]. Therefore, we reasoned that a hematopoietic progeny of mesenchymal cells might become a preferential cellular source for transfusion therapy and cellular therapy.

## Results

### Transformation of HS-5 mesenchymal cells into hematopoietic cells

**Figure 1:**
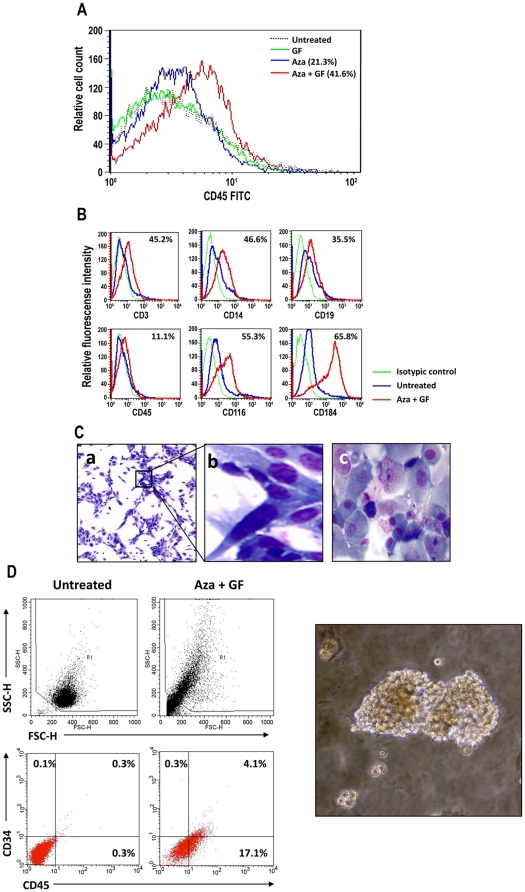
Aza and GF induce hematopoietic cell surface marker expression. (**A**) Incubation of HS-5 cells with Aza induced the expression of CD45 in 21.3% of the cells, whereas incubation with Aza plus GF induced CD45 in 41.6% of the cells. Untreated cells did not express CD45, and incubation of HS-5 cells with GF alone did not affect CD45 expression. (**B**) In another experiment using the same culture conditions, 11.1% of the total population of Aza plus GF–treated HS-5 cells expressed CD45. In addition, a significant percentage of the total population of treated HS-5 cells expressed T-, myeloid-, and B-cell surface markers (CD3, CD14, and CD19, respectively). In addition, the levels of both the GM-CSF receptor (CD116) and the adhesion molecule (CD184) were upregulated. The curves of both untreated and treated cells stained with the isotype antibody overlapped. Therefore only one isotype control curve is depicted. (**C**) Aza plus GF–treated HS-5 cells became round and non-adherent. Untreated HS-5 cells (Write-Giemsa stain, X 100 magnification) show classical morphological features of elongated spindle like cells (a), a X10 magnification of the cells is shown in the insert (b), and panel (c) shows that Aza plus GF–treated HS-5 cells (Write-Giemsa stain of cytospun nonadherent cells, X 1000 magnification) are round, heterogeneous in size and some of them have cytoplasmic granules. (**D**) Left panel: a two-color flow cytometry analysis demonstrated that 4.1% of viable HS-5 cells treated with Aza plus GF (assessed by side (SSC-H) and forward (FSC-H) scatter analysis; top panel) co-expressed CD45 and CD34 antigens. Right panel: a typical BFU-E colony derived from Aza plus GF–treated HS-5 cells grown in methylcellulose.

In order to induce mesenchymal-to-hematopoietic transition, we incubated mesenchymal HS-5 cells with increasing concentrations of Aza at different time points. Our goals were to obtain (1) an optimal reduction in intracellular 5-methylcytidine without compromising cell viability (as assessed by trypan blue staining) and (2) induction of CD45 expression. We found that 2.5 µg/ml Aza (added to HS-5 cells at day 1 and day 4 of culture) reduced the level of intracellular 5-methylcytidine by an average of 21% at day 13 of culture (Figure S1A), and up to 21% of the total cellular population expressed the hematopoietic CD45 antigen (Figure 1A). Furthermore, 47% of the cells expressed the granulocyte-macrophage colony-stimulating factor (GM-CSF) receptor (R)- α CD116, 37% expressed the GM-CSFR-β CD131, and 26% expressed the stem cell factor (SCF) receptor CD117 (Figure S1B). Both growth factors (GFs) have been found to stimulate normal hematopoietic cells and have been used clinically.

We assumed that these GFs might contribute to the transformation of the mesenchymal HS-5 cells into hematopoietic cells, and after determining the optimal dose and schedule, we added 50 ng/ml of both GM-CSF and SCF at days 7 and 10 of culture and harvested the cells for analysis at day 13. Under these conditions, 41.6% of the cells expressed CD45, as compared with 21.3% when the cells were treated with Aza alone (Figure 1A). The transformed cells also expressed different levels of T-cell (CD3), B-cell (CD19), and myeloid cell antigens (CD14, CD64) and myeloperoxydase (MPO) (Figure 1B and S1C). However, CD133, another stem/progenitor cell marker, was not detected in the HS-5 transformed cells and, similarly, not all B-, T-, or myeloid cell markers were upregulated by Aza and GF treatment (Figure S1C). Notably, the MSC-specific markers CD73, CD90 and CD105 were significantly downregulated in the transformed cells (Figure S1D). HS-5 cells treated with Aza plus GF became round and small, as confirmed by morphological (Figure 1C) and side- and forward-scattered flow cytometry analysis (Figure S1E). Notably, a similar percentage of CD45 expression was detected in both the gated small cells and the entire cellular population, suggesting that CD45 expression preceded the morphological changes (Figure S1E).In a two-color flow cytometry analysis of viable cells, 21.2% of the entire Aza plus GF–treated population expressed CD45 and 4.1% expressed both CD45 and CD34 antigens, suggesting that a subpopulation of the transformed cells were early (stem/progenitor) hematopoietic cells. However, the percent of cells expressing both CD45 and CD34 antigens might be underestimated because of quenching [53] (Figure 1D). In other experiments, a significant proportion of Aza plus GF-treated HS-5 cells expressed CD45 (11%–41%; Figures 1A, 1B, and S1E) or co-expressed CD45/CD34 (Figure S1F). Remarkably, Aza plus GF-treated cells formed hematopoietic colonies in semisolid culture medium (Figure 1D). A significant number of colony-forming unit granulocyte-macrophage (CFU-GM), burst-forming unit-erythroid (BFU-E), and CFU-granulocyte-erythroid-macrophage-megakaryocyte (CFU-GEMM) colonies were obtained from 2X10^5^ unfractionated and fractionated nonadherent cells in 5 different experiments (Table S1). As expected, the karyotype of HS-5 cells was complex: 35∼59, XXY, −2, −2, t(2;11), +3, −4, −6, −6, −7, 8, der(8)t(8;14), 10p+, 10q+, −13,der( 13;17), −14,−15, −16, +17, −18, der(18;20), 19q+x2, 20, 21p+, +22, +1∼2mar[cp6]. No additional cytogenetic abnormalities were detected in Aza plus GF-treated HS-5 cells.

### Engraftment of transdifferentiated HS-5 cells in immunodeficient mice

**Figure 2:**
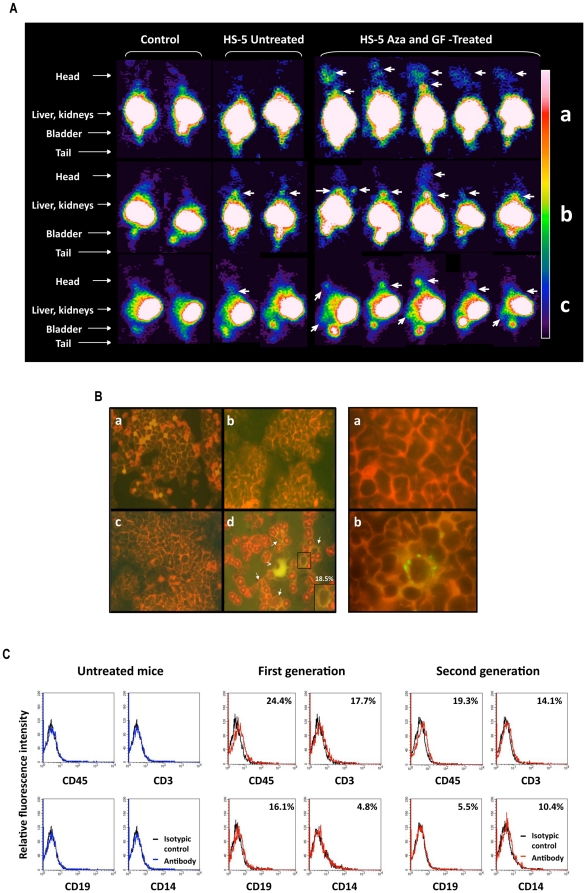
Transformed HS-5 cells engraft and sustain hematopoiesis in NOD-Scid mice. (**A**) Gamma camera imaging studies of NOD-Scid mice. Frontal (a, b) and lateral (c) views 3 days (a) and 3 weeks (b and c) after intravenous injection of 10^5^ untreated or Aza plus GF–treated HS-5 cells, stably transfected with hemagglutinin-A-tagged human somatostatin receptor 2A (SSTR2A) gene. Thirteen MBq (350 µCi) ^111^indium-octreotide was injected intravenously 24 hr prior to imaging. Radioactive signals were detected only in the bladder and kidneys of mice that had not been injected with HS-5 cells (control; first two columns). In contrast, radioactive signals were detected in the calvaria and spine at 3 days (a) and the limbs and/or spine at 3 weeks (b, c) after injection of Aza plus GF–treated HS-5 cells in 8 out of 8 animals (images of 5 animals are depicted). Limb and spine signals were prominently increased after 3 weeks. Weak signals were detected at 3 weeks in the spines of mice that were injected with untreated HS-5 cell (columns 3 and 4; 2 of 4 animals are depicted). (**B**) Left panel: HLA-ABC immunofluorescence of bone marrow smears obtained from NOD-Scid mice 3 weeks after intravenous injection of untreated and Aza plus GF-treated HS-5 cells. Eight mice were injected with Aza plus GF-treated HS-5 cells and 8 mice with untreated HS-5 cells. Bone marrow analysis was performed separately on every mouse. Representative data are depicted. Random fields were scanned and a total of 400 cells, either HLA-positive or -negative, were counted. As shown in panel (d), 18.5% of marrow cells of mice injected with Aza plus GF-treated HS-5 cells were HLA-ABC positive. White arrows point to the positive cells. The insert is a magnification of an HLA-ABC-positive cell. The large cell (arrow head) is a micro-megakaryocyte. No positive staining was detected when slides of marrow cells from Aza plus GF-treated HS-5 cells were stained with the isotype antibody (a) or on slides of marrow cells obtained from mice that were injected with untreated cells (b) or mice that received no injection (c). Right panel depicts a field (X 1000) of HLA-ABC-stained bone marrow cells from a mouse that were injected with Aza plus GF-treated HS-5 cells (right) and a field of HLA-ABC-stained bone marrow cells from mouse that was injected with untreated HS-5 cells (left). (**C**) Flow cytometry analysis of bone marrow cells obtained either from untreated mice, mice that were injected with 10^5^ Aza plus GF-treated HS-5 cells (First generation) or bone marrow cells that were harvested three weeks after injection of 10^5^ first generation bone marrow cells (Second generation). Black line depicts the isotypic control and the percent of antigen-positive cells is depicted in the right upper corners. Similar results were obtained in two different experiments. This analysis was conducted twice with each cohort. Representative results are depicted.

Because a subpopulation of the HS-5 transformed cells expressed the CD34 surface antigen and transformed HS-5 cells formed hematopoietic colonies (Figure 1; Table S1), we wondered whether transdifferentiated HS-5 cells would engraft in NOD-Scid mice. To determine whether these cells home to hematopoietic tissue sites such as the spine and long bones, we used the somatostatin type 2 chimeric gene transfer system [54]. HS-5 cells, stably transfected with hemagglutinin-A-tagged human somatostatin receptor 2A (SSTR2A) gene, were transformed as described above and 1×10^5^ untreated or Aza plus GF-treated HS-5 cells were injected intravenously into 8-week-old NOD-Scid mice 4 hours after they had been exposed to 30 cGy total body irradiation. Three days and 3 weeks later, all mice were injected with 13 MBq (350 µCi) ^111^indium-octreotide and imaged 24 hours later with a gamma camera. As shown in Fig. 2A, three days after injection clear signals were detected in the calvaria of all, and the spine of two, of the mice that were injected with Aza plus GF–transformed HS-5 cells but not in mice that were injected with untreated, stably transfected HS-5 cells. Similarly, no signal was detected in control mice. Significantly stronger signals were detected in the limbs and/or spine of all Aza plus GF–transformed HS-5 cell injected mice three weeks after injection, suggesting that the injected cells engrafted and multiplied. Background signals were seen in the spines of some the mice that were injected with untreated stably transfected HS-5 cells but not in the control mice.

Next, we injected 16 NOD-Scid mice with Aza plus GF–treated or untreated HS-5 cells. Three weeks later, the mice were sacrificed, and their bone marrow was harvested, smeared onto glass slides and stained by immunofluorescence with antibodies to HLA A, B, and C, or suspended in PBS and analyzed by flow cytometry or injected intravenously into another group of 6 sub-lethally irradiated NOD-Scid mice. The second group of mice was sacrificed 3 weeks thereafter, and their bone marrow cells analyzed as described above. The injected Aza plus GF–treated HS-5 cells engrafted in all 8 mice. As shown in Figure 2B, 18.5% of bone marrow cells harvested from mice that were injected with Aza plus GF–treated HS-5 cells expressed HLA ABC, whereas marrow cells of mice injected with untreated HS-5 cells did not expressed these antigens. Furthermore, 24.4% of those cells expressed CD45, 17.7% expressed CD3, 16.1% expressed CD19, and 4.8% expressed CD14 antigen (Figure 2C). Cells expressing CD45, CD3, CD19, CD14, and HLA-ABC antigens were detected, both by flow cytometry and/or immunofluoresecence, in bone marrow cells, and human CD45 and HLA-ABC antigens by flow cytometry in blood cells of all sub-lethally irradiated immunodeficient mice 3 weeks after intravenous injection of 1×10^5^ marrow cells that were harvested from the bone marrow of mice transplanted 3 weeks earlier with Aza plus GF–treated HS-5 cells (Figures 2C and S2), suggesting that a subpopulation of the transdifferentiated cells harbored a second-generation engraftment capacity.

### Transformation of normal bone marrow stroma cells into hematopoietic cells

**Figure 3:**
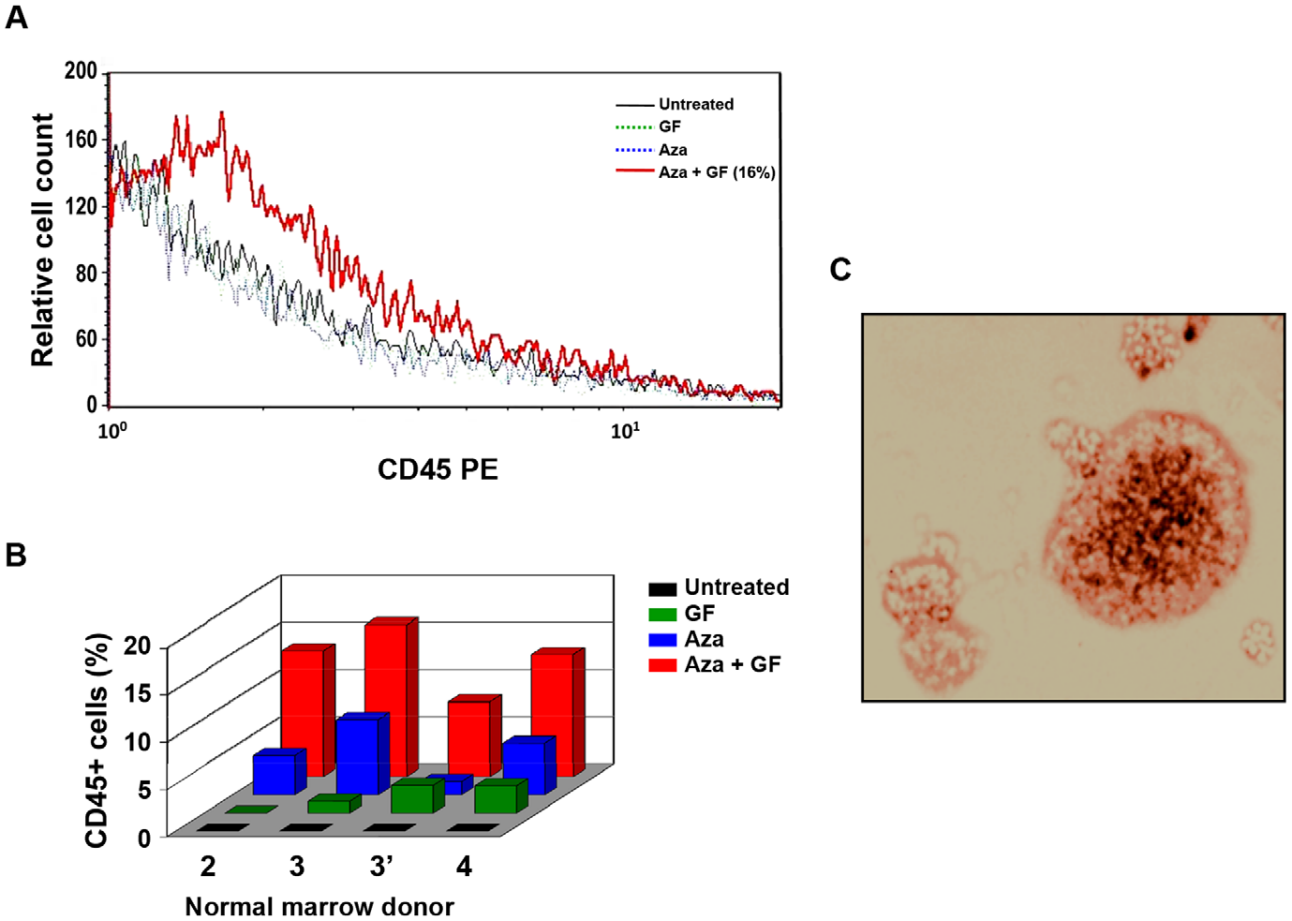
Transdifferentiation of normal marrow MSCs into hematopoietic cells. (**A**) Normal bone marrow MSCs were expanded in culture until all cells were of non-hematopoietic origin. The cells did not express either CD45 or the early stem/progenitor surface marker CD338. Then the cells were incubated with Aza, GF, or both and harvested and stained with rabbit anti-human CD45 antibodies. Only cells that were exposed to both Aza and GF expressed CD45. (**B**) The same experiment was conducted on MSCs derived from bone marrow cells of three additional donors (MSCs of donor 3 were studied twice). In these experiments, CD45 was detected in cells that were exposed to Aza alone. However, significantly higher CD45 expression was found in cells that were treated with Aza plus GF. (**C**) Unlike the elongated untreated MSCs that grew in an adherent monolayer, Aza plus GF-treated normal marrow MSCs gave rise to hematopoietic colony-forming cells. Transformed cells were grown in methylcellulose in the presence of GM-CSF, SCF and erythropoietin and gave rise to CFU-GM, BFU-E and CFU-GEMM colonies. The figure depicts a typical BFU-E colony.

In the previous experiments, we used a bone marrow-derived mesenchymal cell line. Then, we wondered whether Aza together with GF would also transform normal bone marrow MSCs into hematopoietic cells. Again, we performed dose- and schedule-finding studies. We increased the concentration of Aza to 5.0 µg/ml and used a time schedule identical to that used for HS-5 cells. After confirming that the adherent stroma layer contained mesenchymal (CD338+) but not hematopoietic (CD45-) cells, we incubated the MSCs with Aza, GF, or both and demonstrated that Aza plus GF transformed normal bone marrow-derived mesenchymal cells into hematopoietic cells (Figure 3A). Similar experiments were conducted using normal marrow cells from three additional donors. In all experiments, Aza plus GF transformed normal marrow MSCs into hematopoietic cells expressing CD45. In 3 of the 5 experiments, Aza induced low-level CD45 expression, and the effect of GF alone was negligible (Figure 3B). The transformed cells were small, round, mostly non-adherent, and formed hematopoietic colonies when cultured in methylcellulose using a clonogenic assay (Figure 3C; Table S1).

### Gene mRNA and DNA methylation array analysis

**Figure 4:**
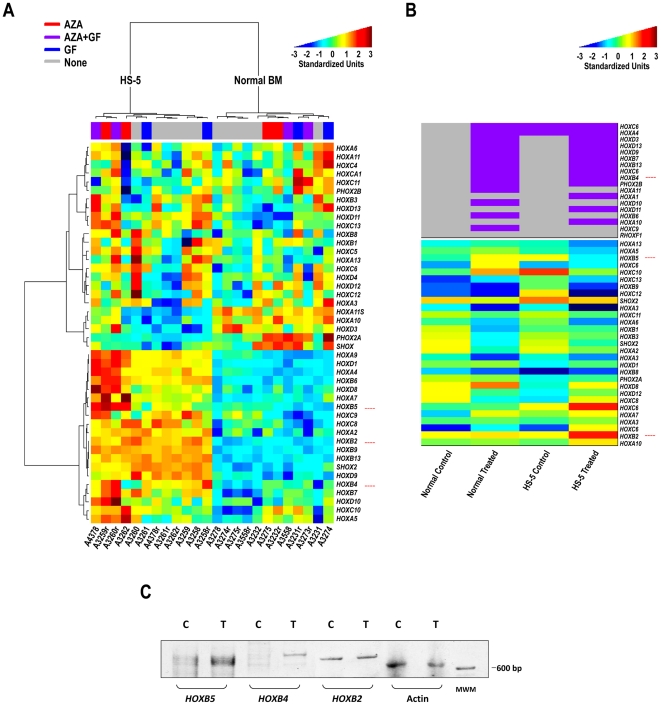
Transdifferentiated mesenchymal cell mRNA and DNA methylation analysis. (**A**) Heatmap of Agilent mRNA expression data. Both rows (genes) and columns (samples) are clustered using the Ward linkage rule and Pearson correlation to define similarity. Some genes were evaluated using 2 or more probes. The dominant signal in the data is the split between HS-5 cells (left branch) and normal bone marrow MSCs (right branch). A secondary signal, particularly prevalent in the HS-5 cells, is driven by the effects of treatment with or without Aza. (**B**) Methylation status of hematopoietic genes. The top portion shows genes that were completely unmethlyated in at least one sample (purple, hypomethylated; gray, methylated). The completely unmethylated genes were omitted before normalization. The bottom portion shows the normalized log ratios from the methylation arrays (blue, weakly methylated; red, strongly methylated). (**C**) RT-PCR studies detected a significant increase in the expression of *HOXB4* and *HOXB5,* but not *HOXB2,* genes following treatment of HS-5 cells with Aza plus GF. C, control (untreated) cells; T, Aza plus GF-treated cells.

Several studies have demonstrated that various genes and transcription factors are capable of altering cell fate ([55], and references therein). Our working hypothesis was that Aza would hypomethylate lineage-restricting genes, enabling GF and culture conditions to bring about transition into cells of the hematopoietic lineage. Therefore, we performed an mRNA array analysis of HS-5 and normal marrow mesenchymal cells following treatment with Aza, GF, or GF plus Aza. Analysis of the array data suggested that of all candidate genes that might be capable of inducing mesenchymal-to-hematopoietic transition, *HOX* genes, particularly those of the *HOXB* family, appeared to be upregulated following Aza treatment. Indeed, a recent gene expression analysis of both mouse and human bone marrow revealed that the majority of *Hox* genes of the A, B, and C clusters are expressed in hematopoietic cells, preferentially in hematopoietic stem cell-enriched populations [56]. Because *HOXB4* and *HOXB5* mRNA levels were upregulated following Aza and GF plus Aza treatment in HS-5 and, to a lesser extent, in normal marrow stroma cells (Figures 4A and S3) and because Aza treatment unmethylated *HOXB4* and hypomethylated *HOXB5* (Figure 4B), we concentrated on these genes. In addition, since the expression of *HOXB2* was slightly upregulated in normal marrow mesenchymal cells (Figure 4A), we included *HOXB2* in the RT-PCR analysis. RT-PCR analysis of Aza-treated and -untreated HS-5 cells confirmed the mRNA array data (Figure 4C). Therefore, we proceeded by testing the effects of these genes.

### Transfection of mesenchymal cells with *HOXB2*, *HOXB4*, and *HOXB5*

**Figure 5:**
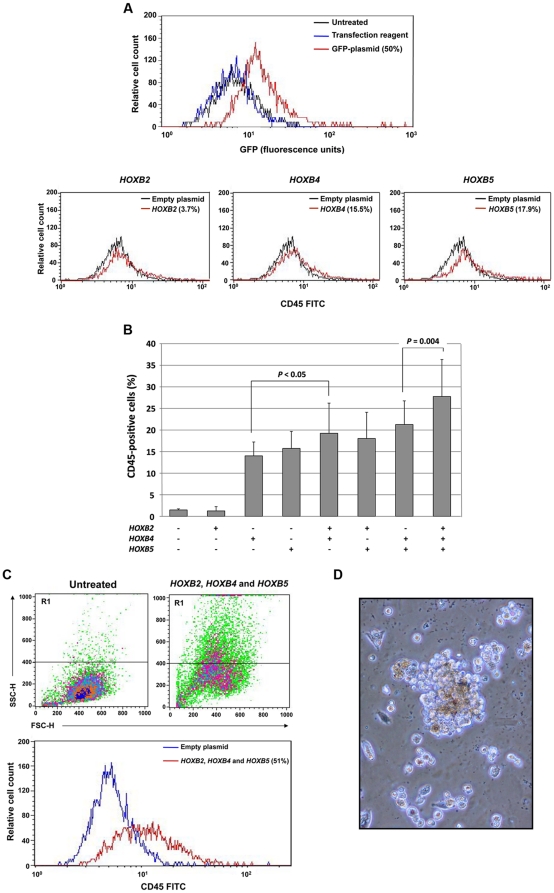
*HOXB* gene transfection transforms HS-5 cells into hematopoietic cells. (**A**) The top panel shows that a 50% transfection efficiency with GFP-tagged plasmid. In all experiments, 40–50% transfection efficiency was obtained. The bottom panel shows that whereas only a marginal change in CD45 expression could be attained by transfection with *HoxB2,* transfection with *HoxB4* and *HoxB5* induced CD45 expression in 15.5% and 17.9% of cells, respectively. (**B**) In three separate experiments, transfection of HS-5 cells with *HOXB4* or *HOXB5*, but not *HOXB2*, induced CD45 expression. However, co-transfection of *HOXB2* with *HOXB4*, or *HOXB2* with *HOXB4* and *HOXB5,* both increased the percentage of cells expressing CD45. The figure depicts the means±S.D. of the percentage of CD45-positive cells. (**C**) Transfection with *HOXB2*, *HOXB4*, and *HOXB5* altered HS-5 cells. The transfected cells became round and small (top panel, right) as compared with untransfected cells (top panel, left) as assessed by side (SSC-H) and forward (FSC-H) scatter analysis, and 51% of the cells expressed CD45 (bottom panel). (**D**) HS-5 cells transfected with *HOXB2*, *HOXB4*, and *HOXB5* formed hematopoietic colonies when grown in the CFU-GEMM colony culture assay. A mixed colony containing erythroid, granulocytic and monocyte-macrophage cells is depicted.

Retroviral overexpression studies in mice suggest that *Hox* genes play a role in hematopoietic stem cell function [reviewed in Ref. [56]]. *HOXB2* plays a role in the pathogenesis of a rare form of retinoic acid-resistant acute promyelocytic leukemia. In this rare leukemia, the promyelocytic leukemia zinc finger (PLZF) gene activates *HOXB2* by binding to its enhancer region [57]. *HoxB4* is expressed in primitive murine and human hematopoietic cells [58], [59], and *HoxB5* is hypomethylated and upregulated in mouse embryo hematopoietic tissue and hypermethylated in adult mice [60], [61]. Taken together, these reports supported our hypothesis that overexpression of these *HOX* genes in mesenchymal cells would enforce a hematopoietic phenotype. We first established experimental conditions to achieve significant transfection efficiency (Figure 5A, top panel). Similar transfection efficiencies were obtained with each gene and gene combination. Then we tested the effects of HOX gene transfection. As shown in the bottom panel of Figure 5A, *HOXB2* induced CD45 expression in only 3.7% of the cells, whereas *HOXB4* and *HOXB5* constitutively induced CD45 expression in 15.5% and 17.9% of the cells, respectively. We then transfected HS-5 cells with 1, 2, or 3 genes and found that *HOXB2* added to the effect of *HOXB4* and *HOXB5* and that transfection with all genes yielded the highest CD45 expression (Figure 5B). Moreover, the cells became round and nonadherent, and a side- and forward-scattered analysis revealed that the cells became smaller (Figure 5C) and a subset of the cells gave rise to hematopoietic colonies in semisolid culture medium (Figure 5D). A significant number of CFU-GM, BFU-E, and CFU-GEMM colonies were obtained from 2×10^5^ unfractionated and fractionated non-adherent cells in 3 different experiments (Table S1).

These results were duplicated using normal marrow-derived mesenchymal cells. As shown in Figure S4, transfection with *HOXB* genes induced morphological and surface marker expression changes similar to those observed in HS-5 cells and to those that occurred with Aza plus GF treatment.

### Transformation of normal skin fibroblasts into hematopoietic cells

**Figure 6:**
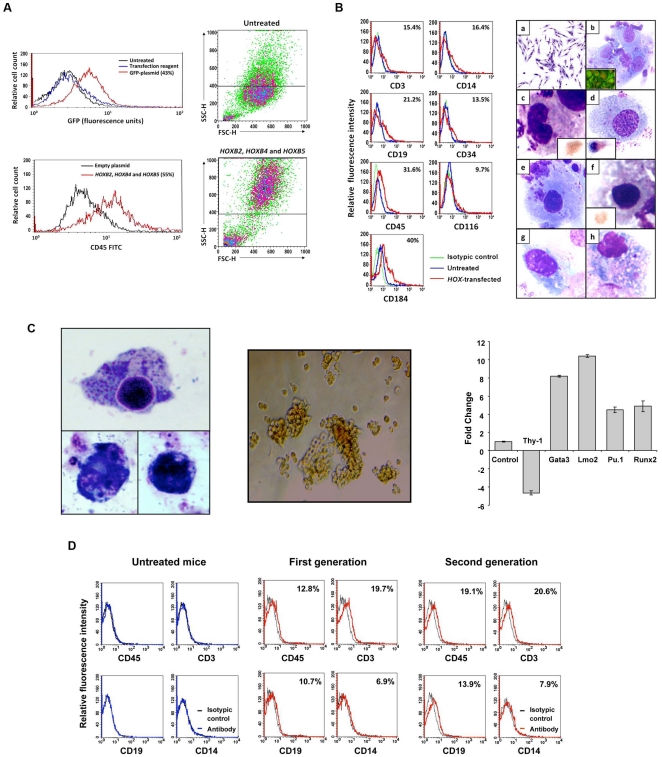
Transformation of skin fibroblasts into hematopoietic cells that engraft in NOD-Scid mice. (**A**) A 43% transfection efficiency of fibroblasts was attained with GFP-tagged plasmid (left, upper panel). Following transfection with *HOXB2*, *HOXB4,* and *HOXB5*, 55% of the cells transformed into CD45-positive cells (left, lower panel). The transformed cells became small and round (right, lower panel) as compared with untransfected skin fibroblasts (right, upper panel). (**B**) Left panel: In a different experiment of co-transfection of skin fibroblasts with *HOXB2*, *HOXB 4,* and *HOXB5* genes, flow cytometry analysis of transfected skin fibroblasts showed that a significant percentage of the total population of the cells expressed the hematopoietic CD45 surface antigen, T-, myeloid-, and B-cell surface markers (CD3, CD14, and CD19, respectively), the immature hematopoietic cell marker CD34, the GM-CSF receptor CD116, and the adhesion molecule CD184 antigens. The percentage of positive cells is depicted in the right upper corner of each figure. The curves of both untreated and transfected cells stained with the isotype antibody overlapped. Therefore only one isotype control curve is depicted. Right panel: Morphological analysis of transfected skin fibroblasts cytospun onto glass slides and stained with Wright-Giemsa demonstrated typical hematologic cell characteristics: (a) cultured untreated skin fibroblasts (X 100 magnification); (b) immature myeloid and lymphoid blasts (insert shows TdT nuclear immunofluorescent staining). The red depicts DNA (propidium iodide stain) and the bright green is nuclear TdT in lymphoblasts (X 1000); (c) mature granulocyte (X 1000). Insert shows an early granulocyte precursor immuno-stained with anti-Kit antibodies (X 1000); (d) a monocyte (X 500) insert shows a monocyte stained with butyrate; (e) a megakaryocyte with platelet membrane demarcations (X 500); (f) normoblasts stained with glycophorine A (X 1000); (g) a lymphocyte (X 1000); (h) a plasma cell (X 1000) with cytoplasmic inclusions (Russell bodies). (**C**) Left panel: skin fibroblasts transfected with HoxB genes differentiate into functional myeloid cells. Three cells (X 100) expressing NBT (dark blue dots) are depicted. Middle panel: skin fibroblasts transfected with HoxB genes form hematopoietic colonies in 4 different experiments. A BFU-E colony grown in the CFU-GEMM colony culture assay is depicted. Right panel: transfection of *HoxB2, HoxB4* and *HoxB5* genes alters skin fibroblast gene expression. Results of qRT-PCR analysis of Thy-1, gata3, Lmo2, Pu.1, and Runx2 gene levels are depicted as fold change (decrease or increase) relative to mRNA levels in empty plasmid-transfected cells. Data from three different experiments are depicted. The means±S.D. of changes in mRNA levels are shown. (**D**) Flow cytometry analysis of bone marrow cells obtained either from untreated NOD-Scid mice, NOD-Scid mice that were injected with 10^5^ *HOXB2-*, *HOXB4-* and *HOXB5*-transfected skin fibroblasts (First generation), or bone marrow cells that were harvested three weeks after injection of 10^5^ first generation bone marrow cells (Second generation). Black line depicts the isotypic control and the percent of antigen-positive cells is depicted in the right upper corners. Human hematopoietic cells were detected in bone marrow cells of all first and second generation mice. The Figure depicts a representative experiment.

To test whether similar results could be obtained with cells of another tissue type, we expanded human skin fibroblasts in culture and, as described above, transfected the cells with *HOXB2*, *HOXB4,* and *HOXB5* genes. Similar to mesenchymal cells, 55% of the transfected fibroblasts expressed CD45 (Figure 6A). The cells became round, non-adherent, and acquired cell surface markers and morphological features typical of immature and mature cells of all hematopoietic lineages. Similar to cells transdifferentiated with Aza plus GF, the *HOX*-transfected cells expressed hematopoietic cell, T-, B-, monocytic, and early hematopoietic cell antigens (CD45, CD3, CD19, CD13, and CD34 antigens, respectively) and, as with Aza plus GF–induced transdifferentiation, the transfected cells expressed upregulated CD116 and CD184 levels. Morphological analysis of cytospins of transfected cells detected myeloblasts, lymphoblasts, immature granulocytes, normoblasts, megakaryocytes, monocytes, lymphocytes, and plasma cells (Figure 6B), and functional studies showed that the *HOXB*-transfected cells expressed NBT and formed hematopoietic colonies (Figure 6C; Table S1). Furthermore, qRT-PCR analysis showed that transfection with *HOXB* genes downregulated the expression of Thy-1, typically expressed at high levels in skin fibroblasts, and upregulated the expression of the hematopoietic genes Gata3, Lmo2, Pu.1, and Runx2 (Figure 6C). To test whether *HOXB*-transformed fibroblasts would engraft in immunodeficient mice, we injected 16 NOD-Scid mice with *HOXB2*-, *HOXB4*- and *HOXB5*-transfected skin fibroblasts as described above. Three weeks later the mice were sacrificed, their bone marrow harvested and analyzed by flow cytometry or suspended in PBS and injected intravenously into another group of 8 sub-lethally irradiated NOD-Scid mice (second-generation). The second group of mice was sacrificed 3 weeks following injection and their bone marrow cells analyzed as described above. As shown in Figure 6D, a significant percent of bone marrow cells obtained from first- and second-generation mice expressed human CD45, CD3, CD19, or CD14 antigens. Remarkably, although 10^5^ unfractionated bone marrow cells were injected, a relatively high percent of human hematopoietic cells of all lineages were detected in second-generation mice.

Then, we attempted to determine whether we could transdifferentiate skin fibroblasts using Aza and GF. Unlike mesenchymal cells, skin fibroblasts required frequent exposure to Aza. Skin fibroblasts were incubated with 5 µg/ml Aza on days 1, 2, 3, and 4, and 50 ng/ml GM-CSF and 50 ng/ml SCF were added on days 4 and 5. The cells were harvested for analysis on day 6 of the initiation of culture. Following treatment, the cells became non-adherent, small, and round, and as shown in Figure S5; 37.7% of the cells expressed CD45 and 26.9% expressed CD34 antigens. The karyotype of Aza plus GF-treated skin fibroblasts was 46,XY, identical to the karyotype of the untreated cells.

## Discussion

To transform mesenchymal cells into hematopoietic cells, we used Aza in combination with GF. Aza is a cytidine analog developed in 1964 as a potentially improved version of the anti-leukemic drug cytarabine [62]. In recent years, Aza has been found to be an active drug in various hematologic malignancies [39] and has been approved by the US Food and Drug Administration (FDA) for the treatment of myelodysplastic syndrome [43]. Aza, an established DNA methyltransferase inhibitor and activator of genes silenced by methylation [36], [37], [38], [39], was found to induce myogenic, adipogenic, and chondrogenic differentiation [63], [64], [65] Aza has been associated with differentiation of cardiomyocytes [66], hepatocytes [67], and epithelia [68], and its effect on myogenic differentiation led to the discovery of the transcription factor MyoD [69]. In our systems, Aza induced morphological changes and CD45 expression. Hematopoietic stem cells and early progenitor cells require a combination of growth factors for self-renewal and differentiation. For example, SCF synergizes with GM-CSF to stimulate hematopoietic stem cells [70], [71], [72]. We found that Aza induced the expression of CD117 (c-Kit; SCF receptor) and both CD116 and CD131 (the α and β components of the GM-CSF receptor) in HS-5 cells. Therefore, we incubated Aza-treated HS-5 cells with these GFs and, as expected, the addition of GM-CSF and SCF increased CD45 expression, as previously found in hematopoietic cells [73]. Furthermore, the combination of Aza plus GF induced the expression of early hematopoietic, T-, B-, and myeloid-cell surface markers. These results agree with previous studies investigating the effects of these GFs [71], [74], [75], [76], [77].

HS-5 cells expressed the chemokine receptor CXCR4 (CD184), and CD184 expression was upregulated upon exposure to Aza plus GF. Previous reports showed that CD184 is expressed by neutrophils, monocytes, T lymphocytes, mature and immature B cells, and CD34+ cells [78], [79] and that adhesion downregulates CD184 cell surface expression [80]. Therefore, it is possible that CD184 levels were upregulated in Aza plus GF–treated HS-5 cells and *HOX*-transfected skin fibroblasts because of transdifferentiation, loss of adherence, or both. Similar to human bone marrow-derived MSCs, HS-5 cells expressed CD10 and CD14 antigens [81], and the levels of those were downregulated following transdifferentiation. Imaging studies demonstrated that intravenously injected transformed human HS-5 cells localized to bones enriched with hematopoietic tissue. Transplantation experiments showed that transformed HS-5 cells engrafted in sub-lethally irradiated immunodeficient mice and that marrow cells from these animals engrafted in the bone marrow of identical sub-lethally irradiated mice. Thus, a subpopulation of Aza plus GF–transformed HS-5 cells possessed hematopoietic stem/progenitor cell characteristics with in vivo self-renewal capacity.

Reproduction of similar results with normal bone marrow MSCs confirmed that this procedure is not cell line restricted and might be clinically applicable. It also raised the possibility that the same molecular mechanism(s) might be recruited to induce mesenchymal-to-hematopoietic transition in both HS-5 and normal marrow MSCs. To elucidate the Aza plus GF transdifferentiation-inducing molecular mechanism, both mRNA and DNA methylation array studies were conducted, and analysis of the data suggested that HOXB transcription factors might be operative in this process. The *Hox* family of homeobox genes are highly evolutionarily conserved genes that encode DNA-binding transcription factors initially identified as regulators of positional identity along the anterior-posterior body axis of animal embryos [82]. *Hox* genes of the A, B, and C clusters are expressed in hematopoietic cells and play a role in hematopoietic stem cell function [56]. Nevertheless, the role of these transcription factors in human hematopoiesis is still poorly understood. *HoxB4* is expressed in primitive murine and human hematopoietic cells [58], [59], and *HoxB5*, expressed in mouse embryonic hematopoietic tissue, is hypermethylated in adult mice [60], [61]. *HOXB2* likely plays a role in human hematopoiesis because its activation contributes to leukemogenesis [57]. Nevertheless, unlike with *HOXB4* or *HOXB5*, transfection of MSCs with the *HOXB2* gene did not significantly increase but rather enhanced CD45 expression induced by *HOXB4* and *HOXB5*. Remarkably, similar to Aza plus GF, transfection of *HOXB2, 4*, and *5* induced mesenchymal-to-hematopoietic transition in both HS-5 cells and normal bone marrow–derived MSCs. These observations prompted the question of whether *HOXB* gene transfection of a non-mesoderm-derived cell could induce hematopoietic transformation.

To address this question, ectoderm-derived skin fibroblasts were chosen. Dermal fibroblasts are easily accessible and expandable in culture, and their morphological features and growth pattern resemble those of MSCs. Skin is the first tissue that was successfully transplanted in humans. Furthermore, fresh or frozen in vitro expanded skin fibroblasts have been successfully used in clinical trials [83], [84], [85], and references therein). Transfection of in vitro expanded human skin fibroblasts with *HOXB2, 4,* and *5* at a transfection efficiency similar to that achieved with MSCs yielded similar results. The fibroblasts transformed into immature and mature hematopoietic cells of all hematopoietic lineages. Similarly, fibroblast-to-hematopoietic cell transition was achieved with Aza plus GF, suggesting that Aza plus GF and *HOX* activate the same molecular mechanism to transdifferentiate skin fibroblasts. Remarkably, *HOXB*-transformed skin fibroblasts also engrafted in immunodeficient mice and exhibited a second-generation engraftment capacity. Whether mesenchymal cells or skin fibroblasts, transformed by Aza plus GF or *HoxB* gene transfection, would rescue lethally irradiated mice and reconstitute their entire hematopoietic system to sustain a normal life span should be determined in future mouse model studies.

The in vivo life span of platelets and granulocytes is short and, unlike red blood cells, platelets and granulocytes cannot be refrigerated for several days. Patients with myelosuppression require frequent transfusion and, as a result, platelets and granulocytes from HLA-unmatched donors have been usually used. Transfusion of a mixed population of mature and immature cells has prolonged the half-life of transfused granulocytes [1]. We obtained both mature and immature hematopoietic cells from transformed mesenchymal and skin cells. Transdifferetiation of MSCs and skin fibroblasts generated a mixed population of mature and immature cells such as megakaryocytes, myeloid progenitor cells and stem/progenitor cells capable of engraftment in a xenograft mouse model, suggesting that the transdifferentiated cells might also be suitable for HSCT. As the number of HSCTs has steadily increased during the past 20 years, a shortage of suitable donors, particularly for African-American and other minority group patients, has limited the number of patients to whom HSCT can be offered. More than 11 million potential stem cell donors have been registered worldwide (www.bmdw.org). Nevertheless, only about 40% of all patients in need of an HSCT from an unrelated donor find a donor with matching HLA-A, -B, -C, and -DRB1 loci at the allele level [86]. Umbilical cord blood may potentially emerge as a stem cell source for patients with hematologic malignancies. However, the clinical use of cord blood cells has been hampered by the relative paucity of stem cells per sample [87], [88], [89] and the lack of an efficient ex vivo stem cell-expansion protocol [90], [91]. Another option, high-dose chemotherapy followed by autologous HSCT, has been used for the treatment of hematologic cancers and several epithelial cancers. Several graft-purging strategies to remove tumor cells yielded modest results [92], [93], however, and as a result, the use of autologous HSCT in cancer patients has gradually declined. Whether transdifferentiated cells from an easily accessible nonhematopoietic cellular source, such as MSCs or skin fibroblasts, might be suitable for HSCT remains to be determined.

Taken together, our data suggest that incubation with Aza and GF, a drug and two hematopoietic growth factors approved for clinical use, or transfection with *HOXB* genes, transforms both human mesenchymal cells and skin fibroblasts into hematopoietic cells. Further studies to determine whether the transformed cells are safe for clinical application are warranted.

## Materials and Methods

### Ethics Statement

This study was approved by the M. D. Anderson Cancer Center Institutional Review Board and the animal studies were approved by the Institutional Animal Care and Use Committee at the M.D. Anderson Cancer Center.

### Cell lines and primary cells

The human marrow-derived HPV-16 (E6/E7)-transformed stroma cell line HS-5 was obtained from the American Type Culture Collection (ATCC; Rockville, MD). HS-5 cells were grown in DMEM α-medium (Gibco, Grand Island, NY) supplemented with 10% bovine calf serum (BCS; Hyclone, Logan UT). Leftover samples of bone marrow aspirates from hematologically normal donors were acquired after obtaining informed consent. Human marrow cells were fractionated using Ficol Hypaque (Sigma, Saint Louis, MO), and the low-density cell fraction was washed in culture medium and incubated in DMEM α-medium supplemented with 20% BCS in T-75 (Becton Dickinson, Franklin Lake, NJ) or T-25 (Corning, Corning, NY) tissue culture flasks at 37°C in a humidified 5% CO_2_ atmosphere. After 48 hours, non-adherent cells were removed and the adherent cells were fed, maintained, and split when they reached 70% confluency for at least 8 weeks, until only adherent mesenchymal stromal cells (MSCs; CD45-, CD14-, CD34-, and CD338 [early stem/progenitor cell marker]-negative cells) were present in culture. Human foreskin fibroblasts were obtained from Lonza Walkersville, Inc. (Walkersville, MD) and maintained in the media that was provided by the supplier. All cells were grown in plastic tissue culture flasks at 37°C in a humidified 5% CO_2_ atmosphere, inspected daily, fed twice weekly, and split once or twice weekly by using 0.25% trypsin (Gibco). Following trypsinization, cells were washed in PBS (Gibco) and maintained in tissue culture medium. In different experiments, cells were incubated in the presence or absence of 2.5 or 5.0 µg/ml 5-azacytidine (Aza), with or without 50 ng/ml GM-CSF and 50 ng/ml SCF, or transfected with *HOXB* genes, as described below. For analysis, cells were trypsinized, washed, and re-suspended in PBS. Bone marrow samples were obtained with informed consent. These studies were performed with the approval of the Institutional Review Board of The University of Texas M. D. Anderson Cancer Center.

### Flow cytometry analysis

Cells were re-suspended in 100 µl PBS and split into duplicate tubes. Twenty microliters antibody or its isotype were added, and the tubes were incubated in the dark at room temperature for 30 min. After incubation, the cells were washed, re-suspended in 500 µl PBS, and analyzed using the FACSCalibur flow cytometer (Becton Dickinson Immunocytometry Systems, San Jose, CA). Data analysis was performed using the software programs provided by the manufacturer. The cytometer was adjusted using the isotypic antibody–treated cells to exclude background fluorescence. A shift from the control curve was calculated as percent shift beyond control. A previously described method was used to detect 5-methylcytidine [94], [95]. In brief, untreated or treated cells were fixed and permeabilized using the BD Cytofix/Cytoperm kit (BD Biosciences, San Diego, CA) in accordance with the manufacturer's instructions. After fixation and permeabilization, the cells were stained with a mouse anti-human 5-methylcytidine antibody (Serotec Inc., Raleigh, NC) for 30 min, washed 3 times with Prewash buffer, and incubated with an RPE-labeled rabbit anti-mouse IgG (Serotec) antibody for 30 min. Then the cells were harvested, washed 3 times in buffer, re-suspended in staining buffer, and analyzed using the FACSCalibur flow cytometer for any decrease in 5-methylcytidine. The appropriate isotype antibodies were used to exclude background staining of untreated and treated cells. For other studies, the following antibodies and their corresponding isotype controls were used: mouse anti-human myeloperoxidase (MPO), mouse anti-human leukocyte antigen (HLA)-ABC, and mouse anti-human CD3, CD8, CD10, CD13, CD14, CD19, CD20, CD33, CD34, CD38, CD41, CD45, CD64, CD 73, CD90, CD105, CD116, CD117, CD131, CD133, CD184 (Becton Dickinson, San Diego, CA), and CD338 (Biolegend, San Diego, CA). In some experiments non-viable cells were excluded using the Miltenyi dead cell removal kit (Miltenti Biotec Inc., Auburn, CA).

### Morphological analysis

Cultured adherent or cytospun non-adherent cells were stained with Write-Giemsa using a standard staining technique. Cytochemistry with terminal deoxynucleotidyl transferase (TdT), butyrate, and glycophorine A and immunocytochemistry staining with anti-Kit antibodies were performed according to the M. D. Anderson Cancer Center standard diagnostic laboratory procedures. Nitroblue tetrazolium (NBT) staining was performed using the NBT reduction kit (Sigma, St. Louis, MO) in accordance with the manufacturer's instructions.

### Colony culture assay

To detect hematopoietic colonies, the colony-forming unit granulocyte-erythroid-macrophage-megakaryocyte assay (CFU-GEMM) was used [96]. Briefly, non-adherent or fractionated (by using the Dead Cell Removal Kit; Miltenyi Biotec, Auburn, CA) 2×10^5^ cells were cultured in 0.8% methylcellulose in Iscove's modified Dulbecco's medium supplemented with 10% FCS, 50 ng/ml SCF (Amgen Inc., Thousand Oaks, CA), 50 ng/ml GM-CSF (Immunex Corp., Seattle, WA), and 1.0 units/ml human erythropoietin (Amgen). One milliliter of the culture mixture was placed in 35-mm Petri dishes in duplicate and incubated at 37°C in a humidified atmosphere of 5% CO_2_ in air. All cultures were evaluated after 14 days for the presence of burst-forming units-erythroid (BFU-E) and colony-forming unit granulocyte-macrophage (CFU-GM). A BFU-E was defined as an aggregate of >500 hemoglobinized cells or three or more erythroid subcolonies, a CFU-GM was defined as a cluster of >50 granulocyte and/or monocyte/macrophage cells whereas a CFU-GEMM as defined as an aggregate of subcolonies containing erythroid, granulocytes and monocyte-macrophage elements.

### Cytogenetic analysis

Untreated trypsinized cells and non-adherent transformed cells were spun down and exposed to hypotonic solution (0.06 M, KC1) for 10 minutes at room temperature. After centrifugation at 1,700 rpm for 5 minutes, the supernatant was discarded, the cell pellet was fixed in a mixture of methanol and acetic acid (3∶1 by volume), and washed three times with a fixative. Fixed cells were dropped on glass slides, and air-dried chromosome preparations were made. Slides were G-banded following the routinely used technique. At least 20 G-banded metaphase spreads from untreated as well as transformed cells were microscopically evaluated.

### Xenograft studies in NOD-Scid mice

All animal work was done in accordance with a protocol approved by the Institutional Animal Care and Use Committee. Eight-week-old female NOD-Scid mice (strain NOD.CB17-PRKDS SCID\J) were obtained from the Jackson Laboratory (Bar Harbor, MI) and maintained in our institutional animal facility. After exposure to 30 cGy sub-lethal whole-body radiation, 1×10^5^ untreated or transformed cells (either Aza plus GF-treated or *HoxB*-transfected) HS-5 cells or skin fibroblasts were injected intravenously. After 3 weeks, imaging studies were performed as described below. The mice were sacrificed and marrow cells were flushed out of both tibias. These cells were analyzed or injected into another batch of sub-lethally irradiated mice that were sacrificed 3 weeks thereafter. The mice were sacrificed in accordance with the experimental protocol when they became moribund or unable to obtain food or water or if they lost >20% of their body weight.

### Mouse imaging studies

HS-5 cells were stably transfected with the hemagglutinin-A-tagged human somatostatin receptor type 2A (*hsstr2*) gene [54]. Stably transfected HS-5 cells were incubated with or without Aza plus GF, as described above, and 1×10^5^ transformed or untransformed cells were injected intravenously into 2-month-old NOD-Scid mice. Twenty four hours before imaging the mice were anesthetized with 2% isofluorane and injected via tail vein with 300 µCi of ^111^indium-octreotide (Mallinckrodt, St. Louis, MO) and anesthetized and imaged the next day using a gamma camera (mCAM; Siemens Medical Solutions, Hoffman Estates, IL) fitted with a medium-energy parallel-hole collimator, as previously described [97]. Planar imaging studies were performed 3 days and 3 weeks after injection of transfected HS-5 cells.

### Immunofluoresecence

Glass slides with cytospun mouse blood or bone marrow cells were stored at 4°C. For analysis, the slides were incubated with normal mouse serum (Sigma, St. Louis, MO) for 1 hr in a humid environment and washed 3 times for 5 min in PBS (Gibco). Then, 20 µl of either rabbit anti-HLA-ABC, -CD45, -CD14, or -CD3 FITC-labeled antibodies or their isotype (Becton Dickinson) were added, the glass slides were covered with a plastic coverslip, and the slides were incubated in the dark at room temperature for 1 hr. After incubation, the slides were counterstained with 0.1% Evan's blue solution (Sigma) washed 3 times in PBS, dried, and mounted with Vectashield mounting media for fluorescence (Vector Laboratories, Burlingame, CA). All slides were scanned, analyzed, and photographed using a fluorescence microscope (Olympus, Center Valley, PA).

### mRNA and methylation gene arrays

Untreated and GF-, Aza-, or GF+Aza-treated HS-5 cells or normal marrow stroma cells were subjected to mRNA expression array analysis, and untreated or Aza-treated HS-5 cells or normal marrow stroma cells were subjected to a DNA methylation array analysis. Hematopoietic gene microarrays were purchased from Agilent Technologies (Santa Clara, CA). RNA was isolated and amplified using standard procedure, and fluorescent cRNA was synthesized from total RNA using the manufacturer's low-input RNA fluorescent linear amplification kit. The kit uses Cy5-CTP (633 nm test channel) and Cy3-CTP (532 nm reference channel) as the fluorescent dyes. One microgram of total RNA was used for the amplification and labeling. For all hybridizations, 750 ng of labeled cRNA sample was used for both Cy5 and Cy3 channels. After hybridization, the arrays were scanned by the Agilent Scanner, producing raw image files. Scanned images were quantified using version 8.1.1.1 of the Agilent Feature Extractor software. Quantification files were loaded into version 2.8.1 of the R statistical software package for processing and analysis (http://www.R-project.org). Median estimates of local background were subtracted from the median estimates of foreground at all spots in each channel of each array, and the data were transformed by computing the base-two logarithm. Control spots were removed before performing within-slide loess normalization between the red and green channels. Between-array normalization was performed by aligning the 75^th^ percentiles. Preliminary analysis to detect dye effects identified approximately 300 spots on the 44 K array that were affected by the dye. In most cases, the dye effect could be attributed to saturation, which was more common in the red channel than in the green channel. Saturated spots were flagged and removed from further analyses. HS-5 and normal marrow MSCs cells were analyzed separately using per-gene two-way ANOVAs with interaction to estimate the effects of Aza treatment or GF treatment. Multiple testing was accounted for by fitting a β-uniform mixture model [98] to the *P*-values measuring the overall significance of the ANOVA models.

The ChiP-GLAS technology for detection of methylated genes (Aviva Systems Biology, San Diego, CA) was used. Briefly, DNA was obtained using standard procedure and split into two samples. One sample was used as the input control (total genomic DNA; green Cy3 channel) and the other for enrichment of methylated DNA (red Cy5 channel). The DNA was biotinylated and annealed with oligos. Each oligo corresponded to one half of each of the 40mers on the microarray. Excess (un-annealed) oligos were removed using streptavidin magnetic beads. Annealed adjacent (paired) oligos were ligated with Taq ligase. Ligated oligos served as templates for amplification with fluorescently labeled primers. Labeled samples were combined and hybridized onto the ChiP-GLAS microarray, scanned, and analyzed. Because standard microarray normalization methods assume that the distributions are the same in the two channels, a multi-step procedure to account for the expected differences was developed. First, the distribution of background intensities over the entire array was tested, and then spots whose foreground intensity was below the 99^th^ percentile of background in each channel were flagged as “undetectable.” In the green channel (total genomic DNA), between 603 and 675 spots were undetectable in at least one array. Of those spots, 547, including all 288 known blanks, were always undetectable. Moreover, 99% of the spots that were undetectable in the green channel were also undetectable in the methylation-enriched red channel. In the red channel, there were between 1240 and 5876 undetectable spots, and these varied widely from one sample to another. The 710 spots that were undetectable in at least one green channel were designated as “Negative Controls,” and spots undetectable in a red channel but not the corresponding green channel were designated as “Hypomethylated.” For normalization purposes, all spots that were Negative Controls or “Unmethylated” on at least one array were removed from consideration. The remaining spots, which gave measurable values in both channels of all arrays, were used for loess normalization between the channels on an array. After normalization, the only evidence of differential methylation within an array came from a relatively small number of spots whose mean log intensity was greater than 10 and which were more than 4 times the median absolute deviation (MAD) away from the identity line. In all cases, these spots were more highly expressed in the methylation-enriched red channel, and so these spots were flagged as “Hypermethylated”.

### Reverse transcriptase polymerase chain reaction (RT-PCR)

Total RNA was extracted from treated or untreated cells using the Total RNA purification kit (Norgen, Thorold, ON, Canada). Reverse transcription reaction was performed with 500 ng of total RNA in a final volume of 20 µl, using M-MuLV Reverse Transcriptase (Roche, Mannheim, Germany) according to the manufacturer-developed procedure. Two microliters of cDNA templates were used for each 50-µl PCR reaction containing 0.5 µM of actin, *HOXB2, HOXB4*, or *HOXB5* gene expression primers that were provided by the manufacturer (Applied Biosystems, Stockholm, Sweden). PCR was performed with Taq DNA polymerase (Roche) in accordance with the manufacturer's instructions. The reaction mixture was heated for 1 min at 94°C and then run for 30 cycles of 94°C for 30 sec, 50°C for 1 min, and 72°C for 1 min, with a final extension of 10 min at 72°C. The PCR products were detected on 2% agarose E-Gel (Invitrogen) containing ethidium bromide. The gels were visualized using a FluorChem 8900 imager (Alpha Innotech Corporation, San Leandro, CA).

### Gene transfection

The GFP-tagged plasmid and DNA transfection-ready system of *HOXB2, HOXB4*, and *HOXB5* was obtained from Origene (Rockville, MD). The DNA was diluted in water in accordance with the manufacturer's instructions. Six-hundred microliters of serum-free Optimem (Gibco) was added to sterile tubes and mixed with 2–6 µl (for each 1 µg of DNA) TurboFectin (Origene) and incubated for 5–10 min. Then, 3 µg DNA was added and the tube was incubated for 15–30 min at room temperature. Tissue culture flasks containing cells at a confluence of 50% to 60% were washed with fresh medium, and the Optimem/TurboFectin/DNA mix was carefully added drop-wise and the flasks incubated for 48 hr. After 48 hr, fresh Optimem/TurboFectin/DNA mix was prepared and added to the cells as described above. After an additional 48 hr, the cells were harvested for further study.

### Quantitative real-time PCR (qRT-PCR)

RNA was isolated using the RNeasy purification procedure (Qiagen, Inc.). RNA quality and concentration were analyzed with a NanoDrop spectrophotometer (ND-1000, NanoDrop technologies, Wilmington, Delaware). Ten micrograms of total RNA was used in one-step RT-PCR (Applied Biosystems, Foster City, CA) with the sequence detection system ABI Prism 7700 (Applied Biosystems) using TaqMan gene expression assay for Thy1, Gata3, Lmo2, Pu.1, Runx2, and 18S (house keeping gene control), according to the manufacturer's instructions. Samples were run in triplicate, and relative quantification was performed by comparing the values obtained at the fractional cycle number at which the amount of amplified target reaches a fixed (C_T_) threshold.

## Supporting Information

Figure S1 Demethylation efficiency and surface marker expression of Aza- or Aza plus GF-treated HS-5 cells. (**A**) To determine demethylation efficacy, methylcytidine levels of untreated and Aza-treated cells were determined by flow cytometry. The figure depicts an experiment in which treatment with Aza reduced methylcytidine levels from 71% to 50%. (**B**) Incubation of HS-5 cells with Aza induced the expression of CD116 (left panel), CD131 (middle panel) and (CD117 right panel). (**C**) Cell surface marker analysis of Aza plus GF-transformed HS-5 cells. As shown in the figure, Aza plus GF treatment downregulated the expression of CD10, CD13, CD20, and CD117 and upregulated the expression of CD34, CD64, and MPO. (D) In addition, Aza plus GF treatment significantly downregulated the expression of the MSC markers CD73, CD90, and CD105. (**E**) Treatment of HS-5 cells with Aza plus GF induced morphological changes. The cells became round and smaller, as assessed by forward- and side-scattered FACS analysis (left panel). Thirty-four percent of the cells became CD45 positive (right upper panel), and 39% of the gated cells (R1, left lower panel) were CD45 positive (right lower panel). (**F**) Co-expression of CD45/CD34 in Aza plus GF-transformed HS-5 cells. Data from 11 different experiments are depicted. The curves of both untreated and treated cells stained with the isotype antibody overlapped. Therefore only one isotype control curve is depicted.
(TIF)

Figure S2 Analysis of bone marrow cells from mice that were injected with HS-5 cells. (**A**) Upper panel: bone marrow slides obtained from mice 3 weeks following injection with untreated (Untreated) or Aza plus GF-treated HS-5 cells (Treated) and immunofluorescently stained with rabbit anti-human CD45, CD14, or CD3 antibodies. Positively stained cells (white arrows) were detected in marrow slides from Treated but not from untreated mice. Bone marrow slides of treated mice did not stain positively with isotype antibodies (not shown). (**B**) Lower panel: bone marrow cells, harvested from NOD-Scid mice 3 weeks following intravenous injection with untreated or Aza plus GF-treated HS-5 cells were injected intravenously into sub-lethally irradiated (30 cGy) NOD-Scid mice. Three weeks later, the mice were sacrificed and their bone marrow was harvested, smeared onto glass slides and stained with anti-HLA-ABC antibodies, and their peripheral blood mononuclear cells were stained with anti-HLA-ABC antibodies and analyzed using flow cytometry. Arrows point to the HLA-ABC-positive cells of the mice that were injected with bone marrow cells of mice treated with Aza plus GF-treated HS-5 cells (Treated). (**C**) Flow cytometry analysis of mononuclear peripheral blood cells obtained from 4 mice that were injected with bone marrow cells of mice treated with Aza plus GF-treated HS-5 cells (second generation). The Figure depicts the percent±S.D. of human CD45-positive and HLA-ABC-positive mononuclear cells in mouse peripheral blood.
(TIF)

Figure S3 Heatmap of Agilent mRNA expression data. Heatmap of Agilent mRNA expression data, including all genes whose standard deviation is at least 0.7. Both rows (genes) and columns (samples) are clustered using the Ward linkage rule and Pearson correlation to define similarity. The dominant signal in the data is the split between HS-5 cells (left branch) and normal bone marrow MSCs (right branch). A secondary signal, particularly prevalent in the HS-5 cells, is driven by the effects of treatment with or without Aza. The list of the analyzed genes is provided at http://bioinformatics.mdanderson.org/Supplements/Datasets/EstrovStemCell.
(TIF)

Figure S4 Transfection with *HOXB2*, *HOXB4,* and *HOXB5* transforms normal bone marrow-derived MSCs into hematopoietic cells. (**A**) Normal marrow MSCs transfected with *HOXB2*, *HOXB4,* and *HOXB5* became round and small (top panel, right) as compared with untransfected cells (top panel, left), and 55% of the cells expressed CD45 antigen. (**B**) Normal marrow MSCs transfected with *HOXB2*, *HOXB4,* and *HOXB5* give rise to hematopoietic colonies when cultured in the CFU-GEMM colony culture assay. A typical BFU-E is depicted.
(TIF)

Figure S5 Aza plus GF-treated skin fibroblasts transform into hematopoietic cells. Skin fibroblasts were incubated with 5 µg/ml Aza on days 1, 2, 3, and 4, and 50 ng/ml GM-CSF and 50 ng/ml SCF were added on days 4 and 5. The cells were harvested for analysis on day 6. As shown in the upper panel, Aza plus GF-treated fibroblasts transformed into small, round cells. Flow cytometry analysis, performed after exclusion of non-viable cells, revealed that 37.7% of the cells expressed CD45 and 26.9% expressed CD34 antigen (lower panel).
(TIF)

Table S1 The types and numbers of hematopoietic colonies grown from transformed HS-5 cells, normal bone marrow MSCs, *HOX*-transfected HS-5 cells, and *HOX*-transfected skin fibroblasts.
(PPTX)
