# Supplementary figures and images for: Transformation of Human Mesenchymal Cells and Skin Fibroblasts into Hematopoietic Cells
Source: PLoS One. 2011 Jun 22;6(6):e21250. doi: 10.1371/journal.pone.0021250 (PMC3120836; doi:10.1371/journal.pone.0021250)

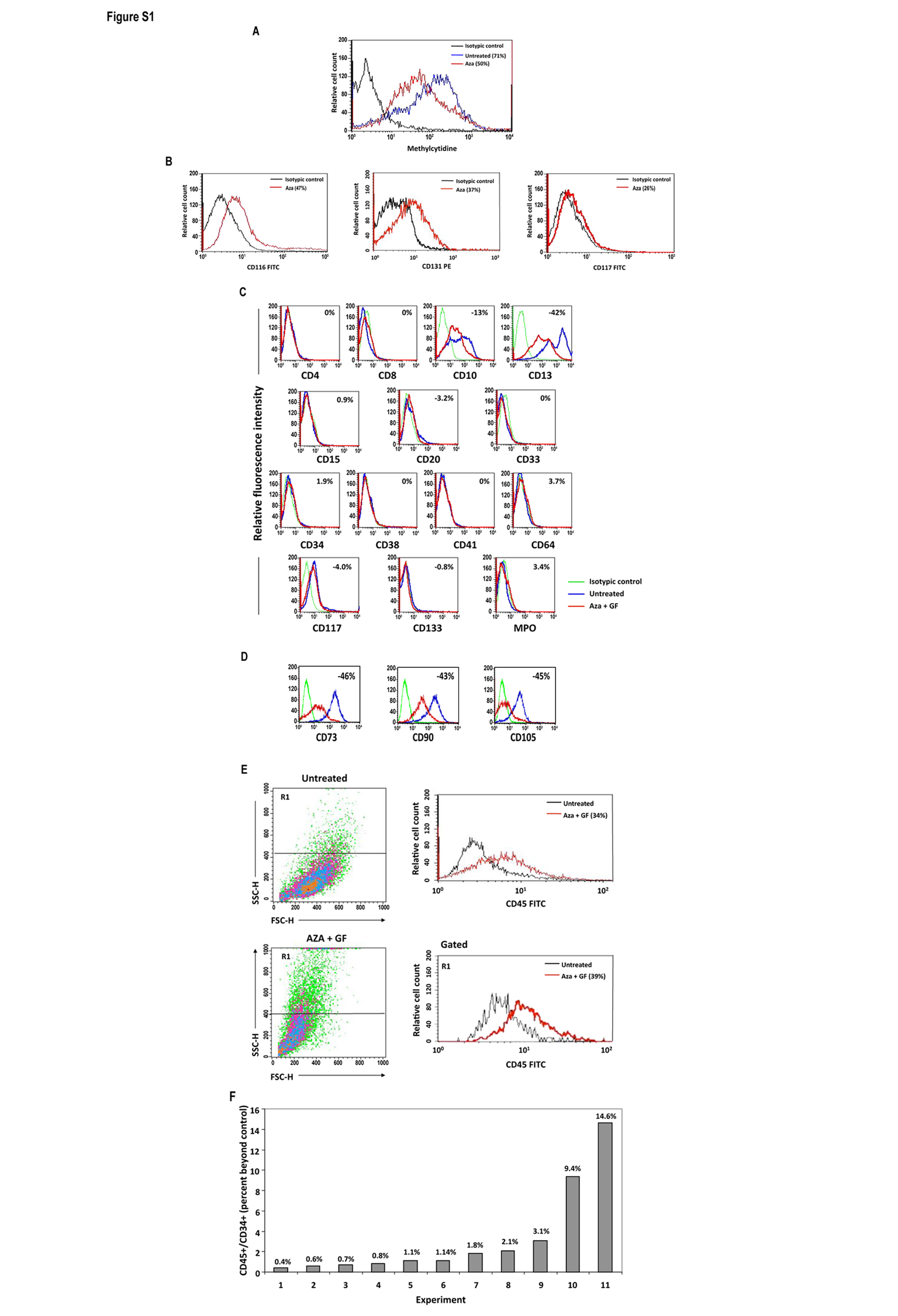

Supplement: Figure S1 — Demethylation efficiency and surface marker expression of Aza- or Aza plus GF-treated HS-5 cells. (A) To determine demethylation efficacy, methylcytidine levels of untreated and Aza-treated cells were determined by flow cytometry. The figure depicts an experiment in which treatment with Aza reduced methylcytidine levels from 71% to 50%. (B) Incubation of HS-5 cells with Aza induced the expression of CD116 (left panel), CD131 (middle panel) and (CD117 right panel). (C) Cell surface marker analysis of Aza plus GF-transformed HS-5 cells. As shown in the figure, Aza plus GF treatment downregulated the expression of CD10, CD13, CD20, and CD117 and upregulated the expression of CD34, CD64, and MPO. (D) In addition, Aza plus GF treatment significantly downregulated the expression of the MSC markers CD73, CD90, and CD105. (E) Treatment of HS-5 cells with Aza plus GF induced morphological changes. The cells became round and smaller, as assessed by forward- and side-scattered FACS analysis (left panel). Thirty-four percent of the cells became CD45 positive (right upper panel), and 39% of the gated cells (R1, left lower panel) were CD45 positive (right lower panel). (F) Co-expression of CD45/CD34 in Aza plus GF-transformed HS-5 cells. Data from 11 different experiments are depicted. The curves of both untreated and treated cells stained with the isotype antibody overlapped. Therefore only one isotype control curve is depicted. (TIF) [file pone.0021250.s001.tif]

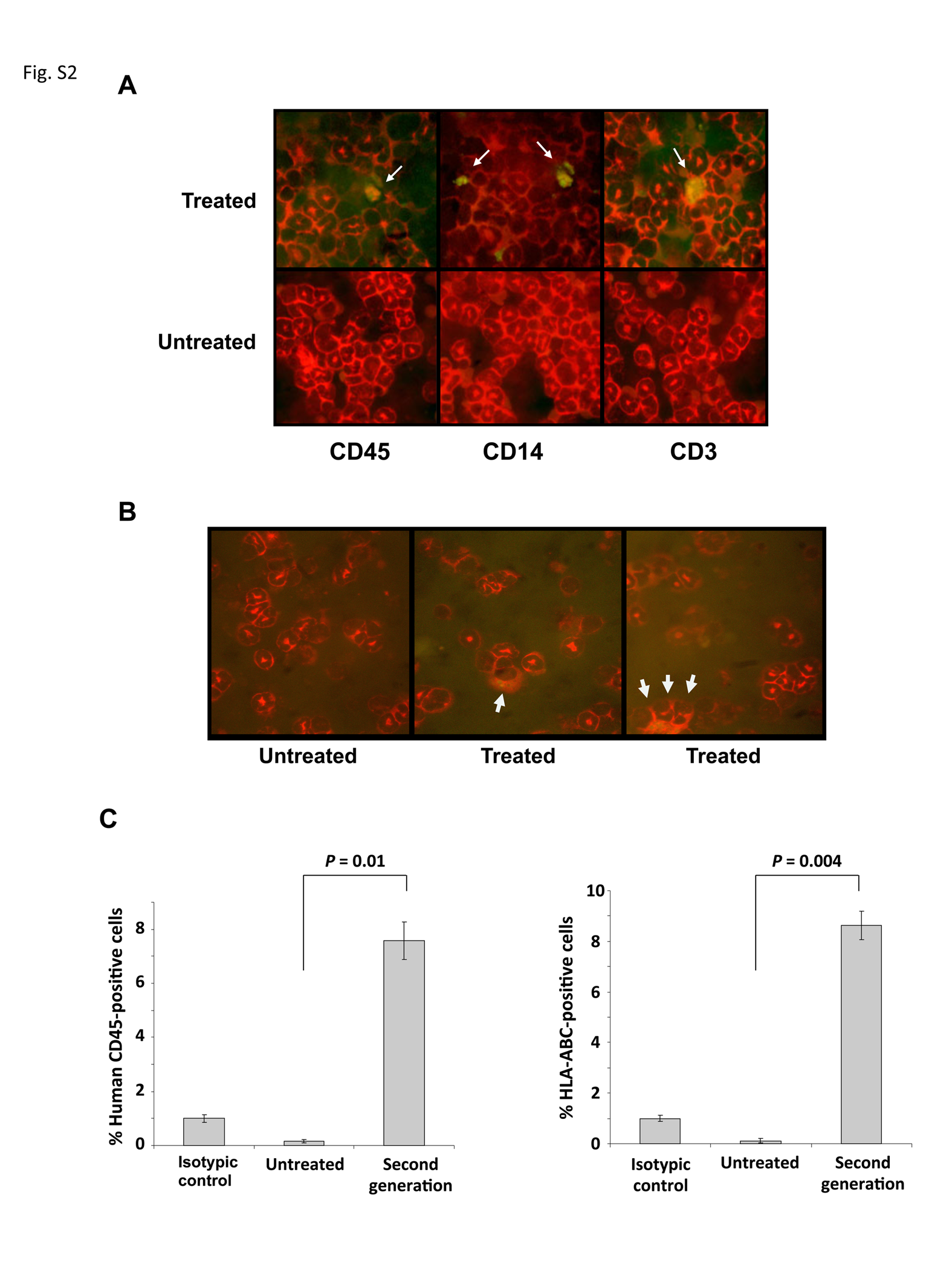

Supplement: Figure S2 — Analysis of bone marrow cells from mice that were injected with HS-5 cells. (A) Upper panel: bone marrow slides obtained from mice 3 weeks following injection with untreated (Untreated) or Aza plus GF-treated HS-5 cells (Treated) and immunofluorescently stained with rabbit anti-human CD45, CD14, or CD3 antibodies. Positively stained cells (white arrows) were detected in marrow slides from Treated but not from untreated mice. Bone marrow slides of treated mice did not stain positively with isotype antibodies (not shown). (B) Lower panel: bone marrow cells, harvested from NOD-Scid mice 3 weeks following intravenous injection with untreated or Aza plus GF-treated HS-5 cells were injected intravenously into sub-lethally irradiated (30 cGy) NOD-Scid mice. Three weeks later, the mice were sacrificed and their bone marrow was harvested, smeared onto glass slides and stained with anti-HLA-ABC antibodies, and their peripheral blood mononuclear cells were stained with anti-HLA-ABC antibodies and analyzed using flow cytometry. Arrows point to the HLA-ABC-positive cells of the mice that were injected with bone marrow cells of mice treated with Aza plus GF-treated HS-5 cells (Treated). (C) Flow cytometry analysis of mononuclear peripheral blood cells obtained from 4 mice that were injected with bone marrow cells of mice treated with Aza plus GF-treated HS-5 cells (second generation). The Figure depicts the percent±S.D. of human CD45-positive and HLA-ABC-positive mononuclear cells in mouse peripheral blood. (TIF) [file pone.0021250.s002.tif]

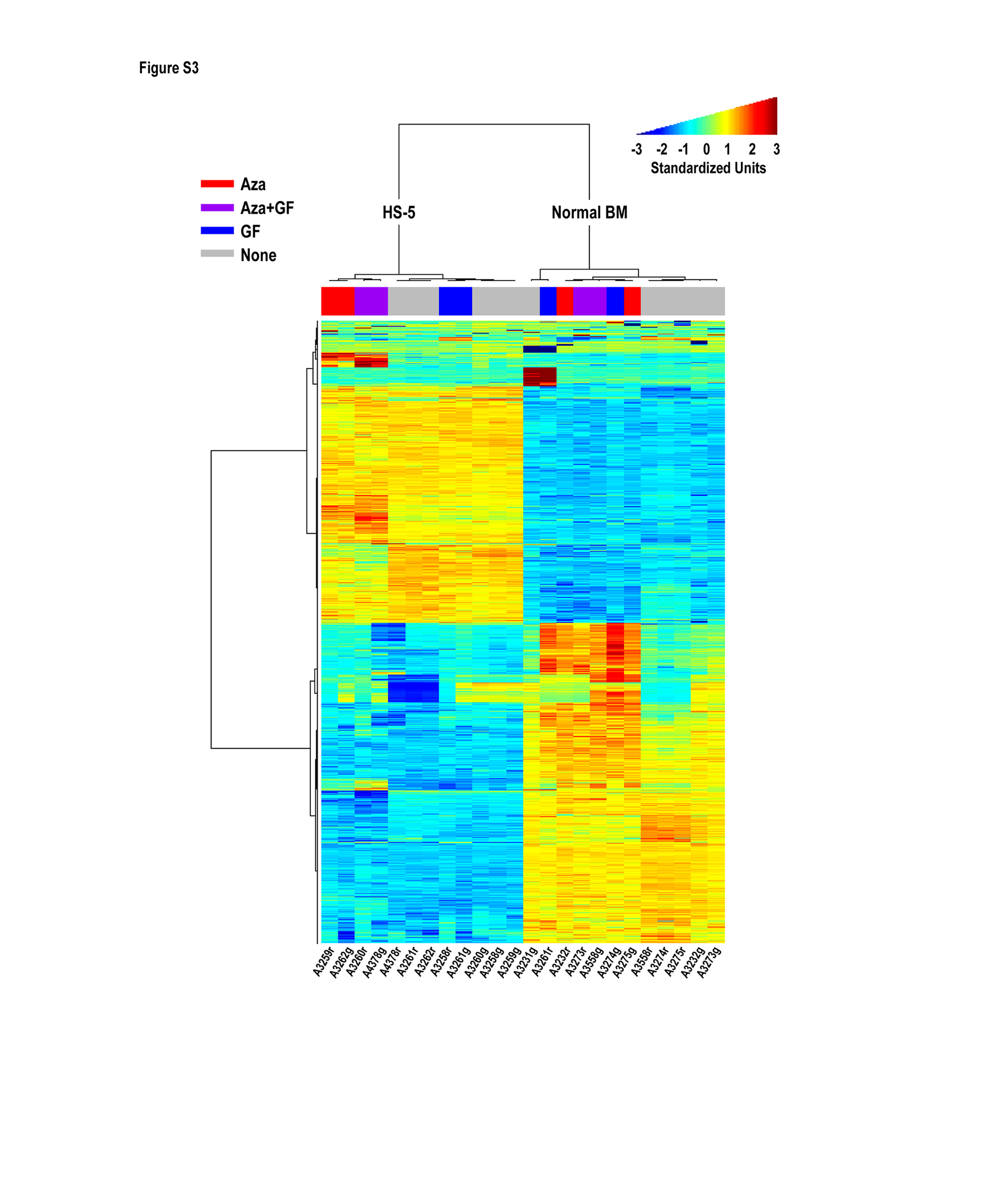

Supplement: Figure S3 — Heatmap of Agilent mRNA expression data. Heatmap of Agilent mRNA expression data, including all genes whose standard deviation is at least 0.7. Both rows (genes) and columns (samples) are clustered using the Ward linkage rule and Pearson correlation to define similarity. The dominant signal in the data is the split between HS-5 cells (left branch) and normal bone marrow MSCs (right branch). A secondary signal, particularly prevalent in the HS-5 cells, is driven by the effects of treatment with or without Aza. The list of the analyzed genes is provided at http://bioinformatics.mdanderson.org/Supplements/Datasets/EstrovStemCell. (TIF) [file pone.0021250.s003.tif]

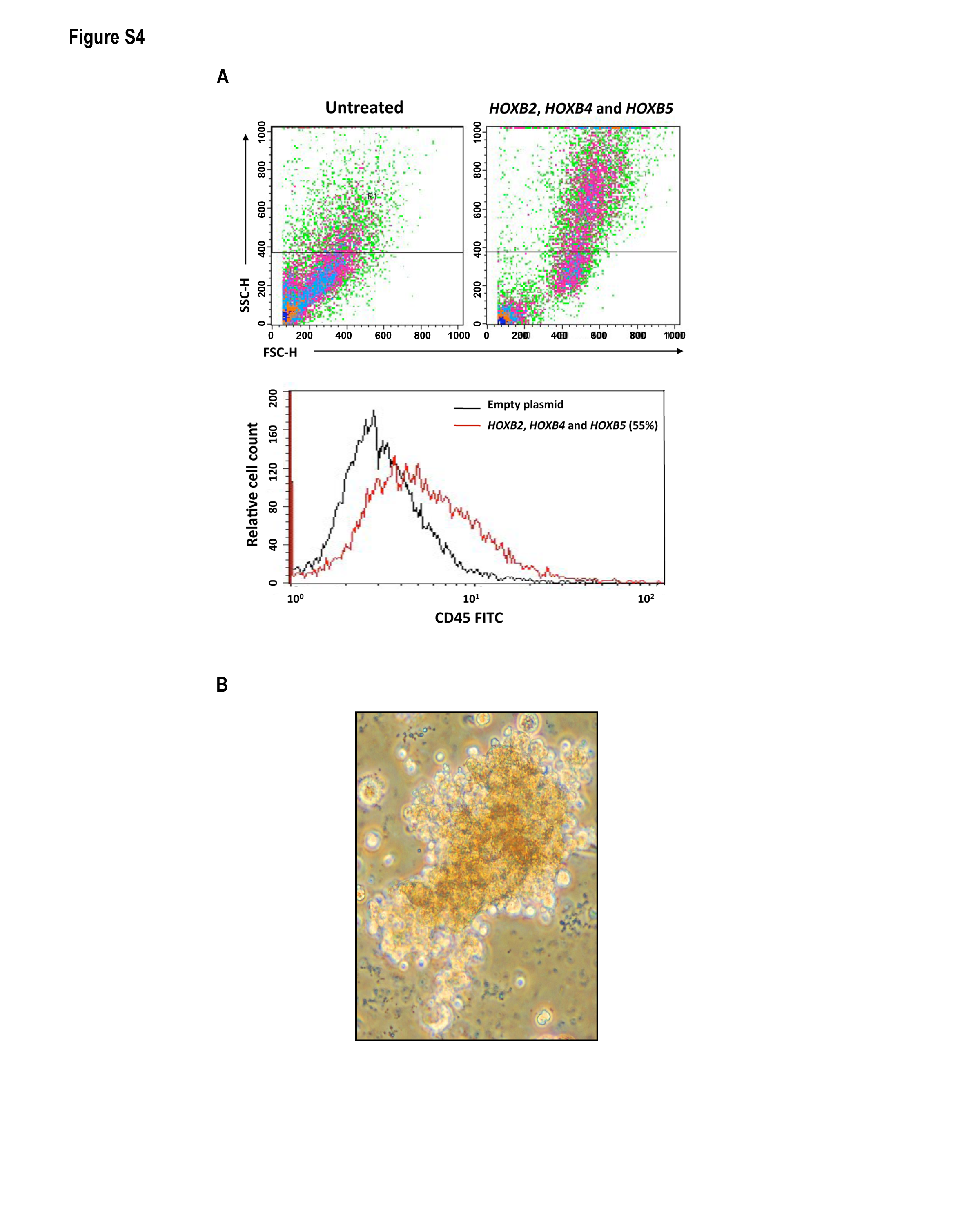

Supplement: Figure S4 — Transfection with HOXB2, HOXB4, and HOXB5 transforms normal bone marrow-derived MSCs into hematopoietic cells. (A) Normal marrow MSCs transfected with HOXB2, HOXB4, and HOXB5 became round and small (top panel, right) as compared with untransfected cells (top panel, left), and 55% of the cells expressed CD45 antigen. (B) Normal marrow MSCs transfected with HOXB2, HOXB4, and HOXB5 give rise to hematopoietic colonies when cultured in the CFU-GEMM colony culture assay. A typical BFU-E is depicted. (TIF) [file pone.0021250.s004.tif]

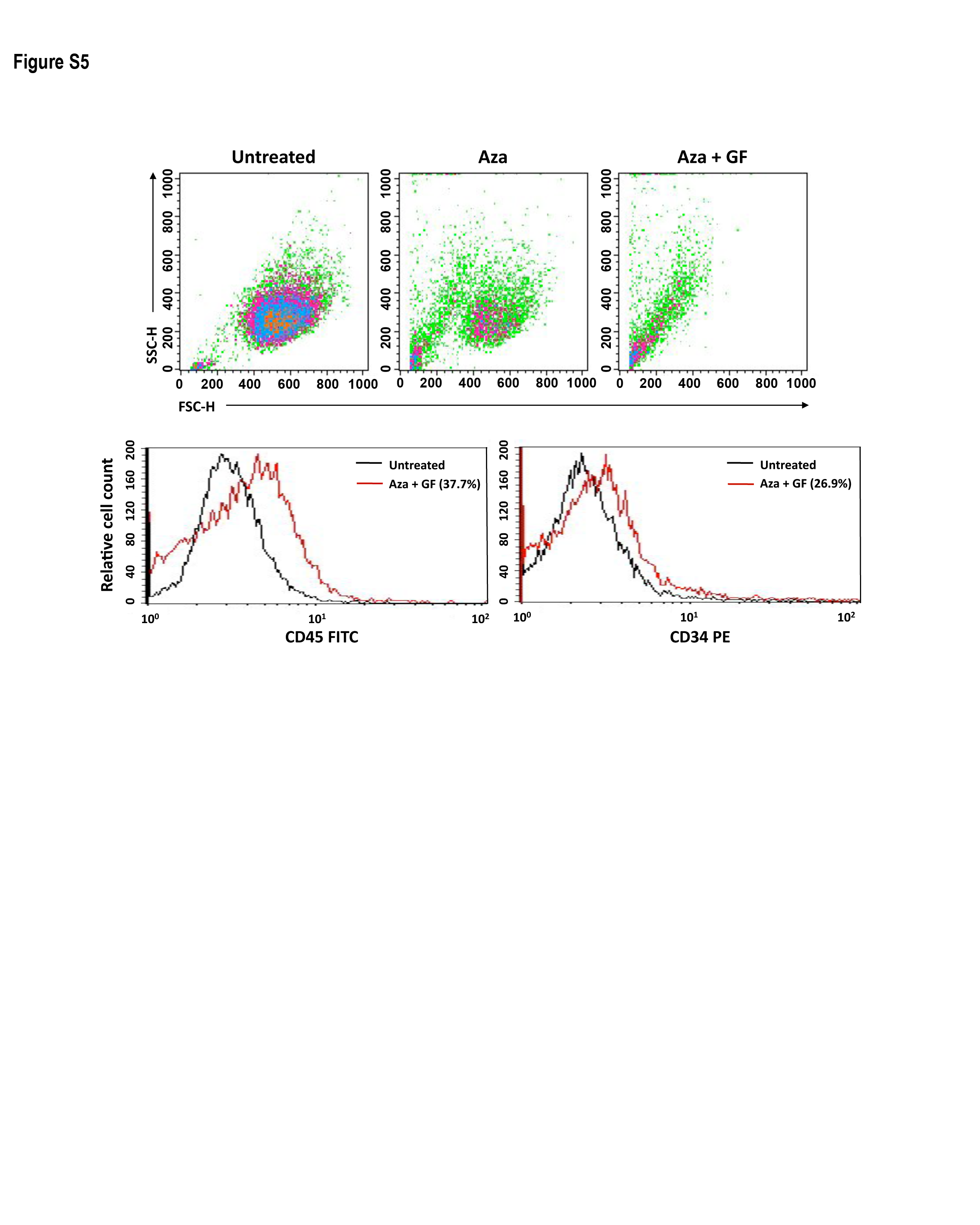

Supplement: Figure S5 — Aza plus GF-treated skin fibroblasts transform into hematopoietic cells. Skin fibroblasts were incubated with 5 µg/ml Aza on days 1, 2, 3, and 4, and 50 ng/ml GM-CSF and 50 ng/ml SCF were added on days 4 and 5. The cells were harvested for analysis on day 6. As shown in the upper panel, Aza plus GF-treated fibroblasts transformed into small, round cells. Flow cytometry analysis, performed after exclusion of non-viable cells, revealed that 37.7% of the cells expressed CD45 and 26.9% expressed CD34 antigen (lower panel). (TIF) [file pone.0021250.s005.tif]
