# Supplementary material for: Transformation of Human Mesenchymal Cells and Skin Fibroblasts into Hematopoietic Cells
Source: PLoS One. 2011 Jun 22;6(6):e21250. doi: 10.1371/journal.pone.0021250 (PMC3120836; doi:10.1371/journal.pone.0021250)
Supplement: Table S1 — The types and numbers of hematopoietic colonies grown from transformed HS-5 cells, normal bone marrow MSCs, HOX-transfected HS-5 cells, and HOX-transfected skin fibroblasts. (PPTX) [file pone.0021250.s006.pptx]

## Slide 1
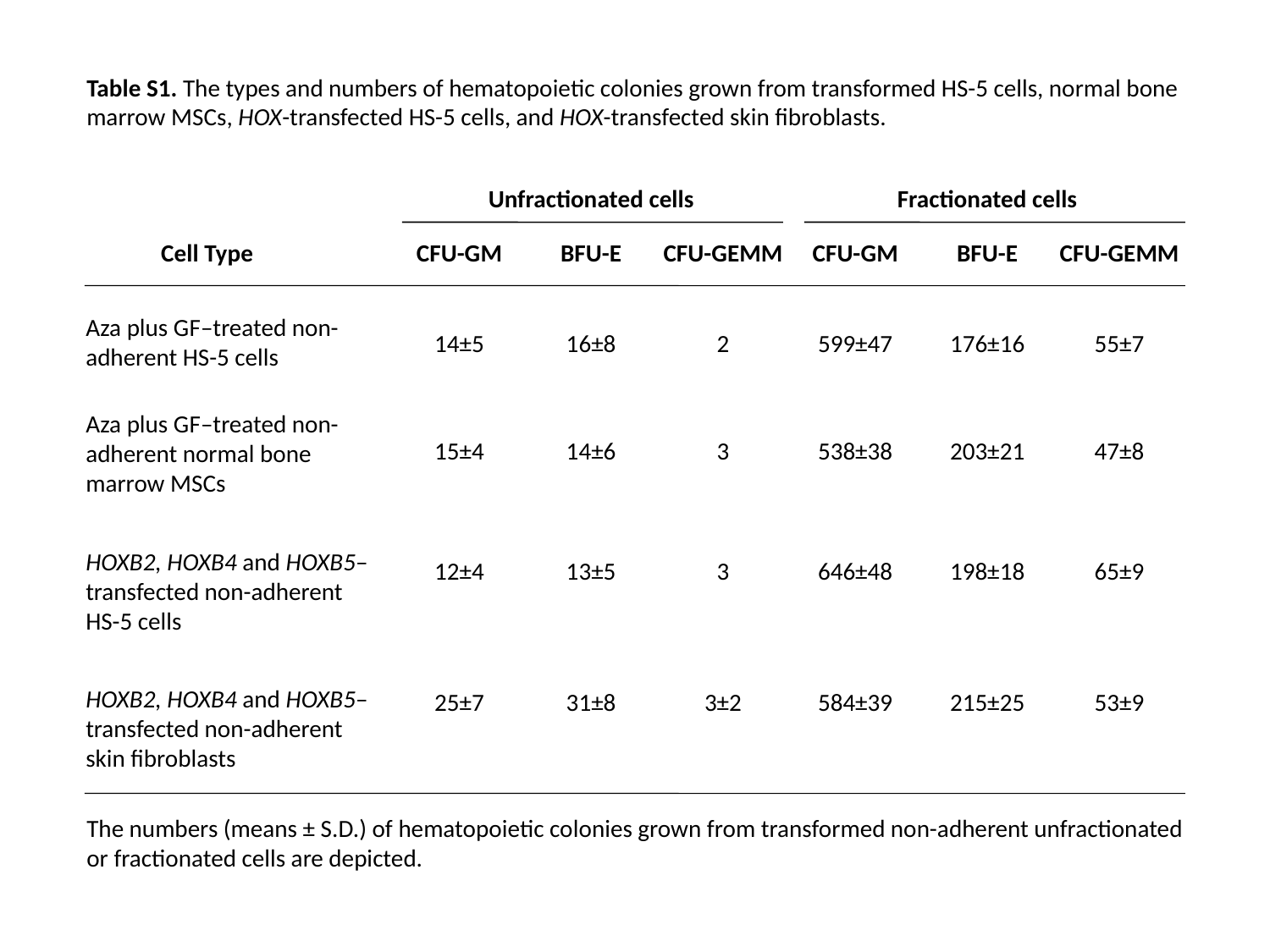

Table S1. The types and numbers of hematopoietic colonies grown from transformed HS-5 cells, normal bone marrow MSCs, HOX-transfected HS-5 cells, and HOX-transfected skin fibroblasts.
Cell Type
Unfractionated cells
Fractionated cells
CFU-GM
BFU-E
CFU-GEMM
CFU-GM
BFU-E
CFU-GEMM
14±5
16±8
2
599±47
176±16
55±7
Aza plus GF–treated non-adherent HS-5 cells
15±4
14±6
3
538±38
203±21
47±8
Aza plus GF–treated non-adherent normal bone marrow MSCs
12±4
13±5
3
646±48
198±18
65±9
HOXB2, HOXB4 and HOXB5–transfected non-adherent HS-5 cells
25±7
31±8
3±2
584±39
215±25
53±9
HOXB2, HOXB4 and HOXB5–transfected non-adherent skin fibroblasts
The numbers (means ± S.D.) of hematopoietic colonies grown from transformed non-adherent unfractionated or fractionated cells are depicted.
